# Role of Dusp6 Phosphatase as a Tumor Suppressor in Non-Small Cell Lung Cancer

**DOI:** 10.3390/ijms20082036

**Published:** 2019-04-25

**Authors:** Verónica Moncho-Amor, Laura Pintado-Berninches, Inmaculada Ibañez de Cáceres, Ester Martín-Villar, Miguel Quintanilla, Probir Chakravarty, María Cortes-Sempere, Beatriz Fernández-Varas, Carlos Rodriguez-Antolín, Javier de Castro, Leandro Sastre, Rosario Perona

**Affiliations:** 1 Department of Experimental Models of Human Diseases, Instituto de Investigaciones Biomédicas C.S.I.C./U.A.M, 28029 Madrid, Spain; Veronica.Moncho-Amor@crick.ac.uk (V.M.-A.); lpintado@iib.uam.es (L.P.-B.); mquintanilla@iib.uam.es (M.Q.); airamcortesempere@hotmail.com (M.C.-S.); bfvaras@iib.uam.es (B.F.-V.); lsastre@iib.uam.es (L.S.); 2 The Francis Crick Institute, London NW1 1ST, UK; 3 Cancer Epigenetics Laboratory, INGEMM, Hospital Universitario La Paz, 28046 Madrid, Spain; inma.ibanezca@gmail.com (I.I.d.C.); rodriguez.antolin.c@gmail.com (C.R.-A.); 4 Biomarkers and Experimental Therapeutics in Cancer, IdiPAZ, 28046 Madrid, Spain; javier.decastro@salud.madrid.org; 5 Departamento de Biotecnología-Instituto de Investigaciones Biosanitarias, Facultad de Ciencias Experimentales, Universidad Francisco de Vitoria, 28223 Madrid, Spain; emvillar@iib.uam.es; 6 Bioinformatics, Francis Crick Institute, 1 Midland Road, London NW1 1AT, UK; probir.chakravarty@crick.ac.uk; 7 CIBER de Enfermedades Raras (CIBERER), 28029 Madrid, Spain; 8 Department of Oncology, Hospital Universitario La Paz, 28046 Madrid, Spain

**Keywords:** *DUSP6*, MKP3, tumor suppressor, ERK1/2, ERK5, tumorigenesis, EMT

## Abstract

DUSP6/MKP3 is a dual-specific phosphatase that regulates extracellular regulated kinase ERK1/2 and ERK5 activity, with an increasingly recognized role as tumor suppressor. In silico studies from Gene expression Omnibus (GEO) and Cancer Genome atlas (TCGA) databases reveal poor prognosis in those Non-small cell lung cancer (NSCLC) patients with low expression levels of *DUSP6*. In agreement with these data, here we show that *DUSP6* plays a major role in the regulation of cell migration, motility and tumor growth. We have found upregulation in the expression of several genes involved in epithelial to mesenchymal transition (EMT) in NSCLC-*DUSP6* depleted cells. Data obtained in RNA-seq studies carried out in *DUSP6* depleted cells identified EGFR, TGF-β and WNT signaling pathways and several genes such as *VAV3, RUNXR2, LEF1, FGFR2* whose expression is upregulated in these cells and therefore affecting cellular functions such as integrin mediated cell adhesion, focal adhesion and motility. Furthermore, EGF signaling pathway is activated via ERK5 and not ERK1/2 and TGF-β via SMAD2/3 in *DUSP6* depleted cells. In summary *DUSP6* is a tumor suppressor in NSCLC and re-establishment of its expression may be a potential strategy to revert poor outcome in NSCLC patients.

## 1. Background

Lung cancer is the leading cause of cancer deaths worldwide. Non-small-cell-lung cancer (NSCLC) represents about 85% of all lung cancers. Despite advances in early detection and standard treatment, NSCLC is often diagnosed at advanced stages with a poor prognosis [1]. Treatment in those patients is focused in preventing relapses and cancer spread for as long as possible, by using a combined cisplatin-based chemotherapy the only treatment proven to increase overall survival in patients with advanced NSCLC [2]. 

Most lung adenocarcinomas harbor mutations in *KRAS* gene (32%) and epidermal growth factor receptor (EGFR) (11%), resulting in overactivation of the RAS-RAF-MEK-ERK pathway [3,4]. Therefore, ERK signaling appears important or critical in at least 30–50% of NSCLC. ERK1/2 is activated by dual threonine and tyrosine phosphorylation of a TxY (threonine-x-tyrosine, TEY) motif by the mitogen-activated protein kinases (MAPKs), mitogen-activated protein kinase kinase 1 (MEK1) and mitogen-activated protein kinase kinase 2 (MEK2), which in turn are activated by oncogenic drivers RAF-RAS. ERK5 is a relatively recently identified MAPK and displays different functions from MAPK family members. ERK5 activation mediated by RAS [5] has been associated with a diverse range of cellular processes including cell proliferation, migration, survival and angiogenesis [6]. Besides, activation of ERK5 in fibroblasts can lead to changes in the organization of actin cytoskeleton, including a loss of stress fibers [7] and formation of invasive adhesion structures termed podosomes [8].

The intensity and long-lasting effect of ERK1/2 signaling is regulated by a family of dual-specific mitogen activated protein (MAP) kinase phosphatases (DUSPs), including both cytoplasmic (DUSP6, 7 and 9) and nuclear DUSPs (DUSP5). DUSPs act differently and may even play opposing roles in various cancers depending on tumor types and progression state of the disease [9]. MAP kinase phosphatase 6/DUSP6 in particular, has been shown to act as a negative feedback regulator for ERK1/2 and ERK5, inhibiting the mitogenic response mediated by those kinases. DUSP6 is functionally involved in suppressing tumor progression in pancreatic, ovarian and lung cancers [10]. Down-regulation of its expression is observed in primary ovarian cancer. In lung cancer, DUSP6 is progressively lost, as tumor grade increases. In addition, the tumor suppressive effects of DUSP6 have been demonstrated both in in vivo and in vitro assays, in ESCC and NPC. Moreover, it is suggested to modulate epithelial-mesenchymal transition (EMT) properties, being associated with loss of invasiveness [10]. 

Here we investigate the role of DUSP6 in NSCLC tumorigenesis and EMT-associated properties. To gain insight into the cellular signaling pathways involving DUSP6 actions in NSCLC, we have performed RNA-seq in combination with functional depletion of *DUSP6* by shRNA. We first obtained a differential expression profile of genes regulated by DUSP6 in NSCLC cells, suggesting its role in focal and integrin-mediated adhesion and the regulation of EGF and TGF-β signaling pathway. We then functionally tested the lack of adhesion in *DUSP6* silenced cells and demonstrated that ERK5 and SMAD proteins are involved in the progression of this tumorigenic phenotype. All these data support the potential role of *DUSP6* as a tumor suppressor gene in non-small cell lung cancer. 

## 2. Results

### 2.1. DUSP6 Acts as a Tumor Suppressor Gene in Lung Adenocarcinoma

**Figure 1:**
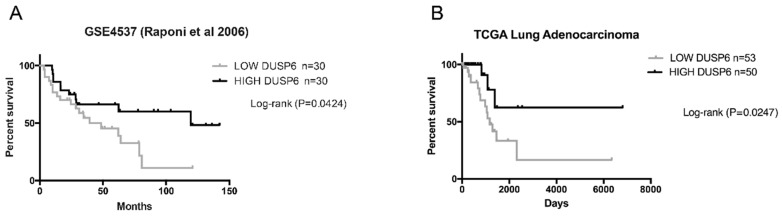
Kaplan-Meier survival analysis according to *DUSP6* gene expression in Lung Adenocarcinoma. (**A**) Kaplan-Meier plots based on GSE4537 dataset in a cohort of 60 human lung adenocarcinoma showing that patients with low *DUSP6* expression (grey) had statistically significant decreased overall survival and disease-free survival compared with patients high *DUSP6* expression level (black). (**B**) Kaplan-Meier plot of *DUSP6* expression in human LUAD based on TCGA dataset in a cohort of 103 patients.

To investigate the role of *DUSP6* in lung cancer, we analyzed the expression profile of DUSP6 from publicly accessible large datasets of NSCLC, deposited in Gene Expression Omnibus (GEO) and the two different types of lung cancer LUAD (Lung Adenocarcinoma) and LUSC (Lung Squamous Cell Carcinoma), deposited in The Cancer Genome Atlas (TCGA) databases. Low and high *DUSP6* expression tertile group data of patients from both datasets were assessed on a survival curve. Importantly, in the GSE4537 dataset [11] low *DUSP6* expression showed to be associated with poor outcomes of lung adenocarcinoma, including decreased overall survival of the patients (long rank *p* = 0.0424) (Figure 1A). Furthermore, the results in the LUAD TCGA cohort confirmed those obtained with the GSE4537 dataset (Figure 1B). In contrast we could not detect a correlation between low *DUPS6* expression and poor outcome in the LUSC TCGA cohort. Low *DUSP6* expression in lung adenocarcinoma patients diminishes survival but not in LUSC patients. 

### 2.2. Inhibition of DUSP6 Expression Induces Changes in Cell Morphology, Anchorage, Motility and Tumorigenesis in Non-Small Cell Lung Cancer Cells

**Figure 2:**
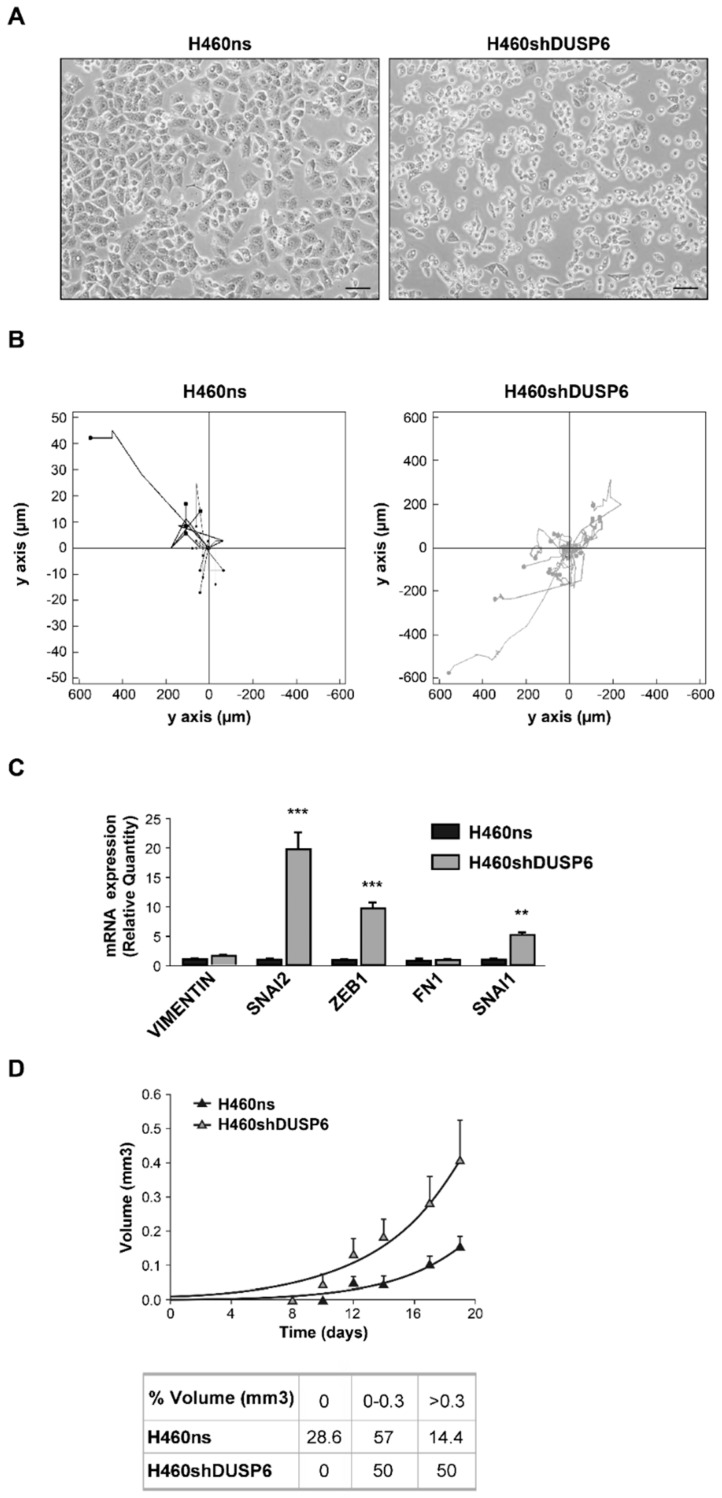
Lack of *DUSP6* expression induces changes in morphology, anchorage, motility and tumorigenesis. (**A**) 20× representative phase contrast photomicrographs of H460 cells infected with non-silencing vector (H460ns) and infected with construct 6 of *DUSP6* viral shRNA (H460shDUSP6). Bar scale corresponds to 20 m. (**B**) Spatial plot of cell movement from H460ns or H460shDUSP6 cells. The assay was recorded during 24 h and images were captured every 30 min. (**C**) Quantitative PCR validation of genes involved in epithelial to mesenchymal transition (*VIMENTIN, SNAI2, ZEB1*, Fibronectin1 (*FN1), SNAI1).* Data were normalized to *β-actin* levels and are shown as mean ± s.d. of three independent experiments. Statistical significance indicated by asterisks considering at ** for *p* < 0.01 and *** for *p* < 0.001. (**D**) Graph indicates tumor volume recorded every day after (each point represented mean ± s.d.); table indicates the percentage of emergent tumors in each group and the volume.

To analyze which is the role of *DUSP6* in NSCLC, we selected the H460 cell line that belongs to this type of lung cancer. The first approach was the depletion of *DUSP6* gene expression by using shRNA lentiviral vectors. Several constructs were tested and two clones 6 (H460shDUSP6) and 9 (H460shDUSP69) were chosen displaying minimum expression of the gene (Supplementary Figure S1) for in vitro assays. We observed that control cells or cells infected with non-silencing vector (H460ns) spread on the culture plate surface shortly after seeding, while *DUSP6* depleted cells were more rounded, smaller and appeared to adhere more loosely to the culture dish (Figure 2A). 95% of *DUSP6* depleted cells (10 fields were quantified) showed round morphology while only 10% of H460ns cells showed a normal flat morphology. In addition, when we treated the cells with low trypsin concentrations, H460shDUSP6 cells detached from the culture-treated plates earlier than control cells, presumably due to their decreased adhesive capability (see Figure 2A, none bars).

In view of this change in morphology and adhesion, we investigated whether cell motility was regulated by *DUSP6*. Individual cell migration was therefore monitored in *DUSP6* depleted versus control cells. We observed that H460shDUSP6 cells were more motile (379 µm ± 73 versus 25 µm ± 14, *p* = 0.05) and also faster (0.26 µm/min ± 0.05 versus 0.02 µm/min ± 0.007 *p* = 0.05) than control cells (Figure 2B). *DUSP6* depleted cells displayed a random path length directionality. However in vitro wound healing assay showed a random pattern of migration in H460shDUSP6 cells compared to the control cells, which pulled in one direction to heal the wound (Supplementary Movie S1).

We observed that *DUSP6* depleted cells lose their morphology, cell-cell adhesion, cell-matrix adhesion and gain migratory properties similar to an epithelial to mesenchymal transition (EMT) process. Hence, we decided to study the expression of a set of genes involved in *EMT; VIM, SNAI2, ZEB1, FN1, SNAI1*. In H460shDUSP6 cells showed increased expression levels of *SNAI1, SNAI2* and *ZEB1* genes compared to control cells (Figure 2C). 

Finally, to evaluate the DUSP6 action in tumorigenesis, both H460ns (control cells) and H460shDUSP6 cells were injected into the flanks of nude mice. Tumors derived from H460shDUSP6 cells grew at notably higher rates than tumors induced by H460ns cells in nude mice (*p* < 0.0001, Figure 2D). The tumor doubling time of H460shDUSP6 cells (3.657) was higher than that of H460ns cells (3.055). 

### 2.3. Gene Expression Profiles of H460 DUSP6 Depleted Cells

Based on the changes of cell morphology and lack of anchorage, we compared gene expression profiles between *DUSP6* silenced and control in H460 (NSCLC) cells by RNA sequencing.

**Figure 3:**
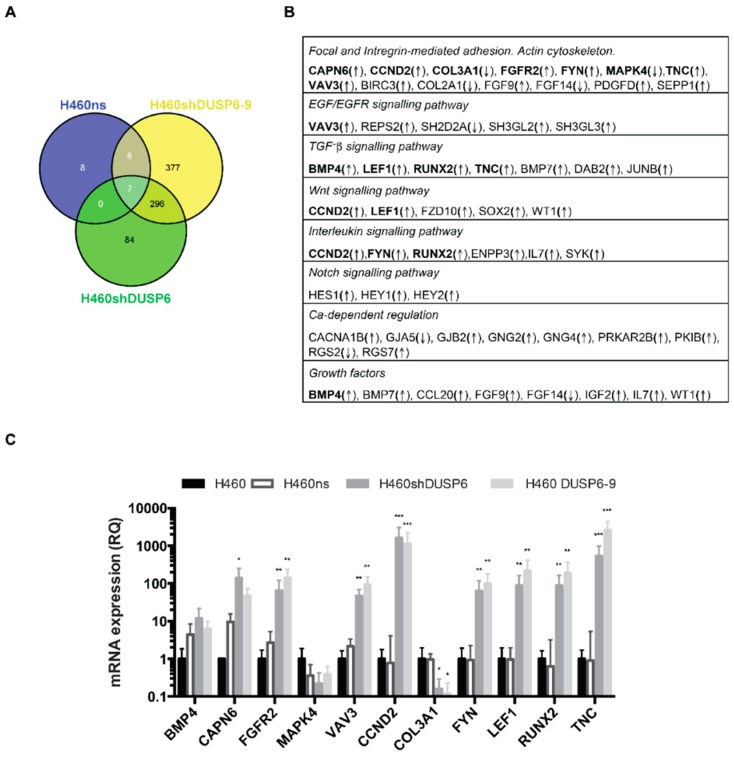
Gene expression profiles of DUSP6 depleted H460 cells. (**A**) Venn diagram comparing the genes regulated in H460ns and the two clones of silenced DUSP6, H460shDUSP6-6 and H460shDUSP6-9 cells. (**B**) Table of statistically significant expression genes involved in pathways regulated by DUSP6 in H460 cells. (**C**) RNA-seq validation by qRT-PCR of selected genes (genes indicated in bold in Figure 3B). Data indicated as RQ (relative quantification) were normalized to β-actin levels and are shown as mean ± s.d. of three independent experiments. Statistical significance indicated by asterisks considering at * *p* < 0.05. ** for *p* < 0.01 and *** for *p* < 0.001.

The expression of 686 or 387 genes was significantly affected in cells infected with a *DUSP6* silencing vector compared to cells that were respectively non-infected or infected with a non-silencing vector. In contrast, expression of only 21 genes was modified between non-infected H460 and cells infected with a non-silencing vector H460ns. The expression of 296 genes was commonly regulated in the maximum inhibition *DUSP6* pair clones (H460shDUSP6-6 and H460shDUSP6-9) compared to control, with 266 upregulated and 30 genes downregulated (Figure 3A). The functional significance of this change in the gene expression pattern was approached by Gene ontology analysis. Several of the regulatory pathways differentially regulated upon *DUSP6* silencing were involved in cell-cell and cell-substrate adhesion, belonging to the focal and integrin-mediated adhesion gene family and actin cytoskeleton regulation (Figure 3B), in agreement with the observed increased motility in silenced cells (Figure 2B). Other signaling pathways were also affected such as EGF, TGF-β and Notch mediated pathways. The differences in expression levels detected by RNA-seq were validated by quantitative RT-PCR for 11 genes (indicated in bold face in Figure 3B) (Figure 3C). Similar results were obtained when we used *GAPDH* as control for gene expression (not shown). In addition, the differential expression in *DUSP6* depleted cells were validated in A549 cells, 8 out of 11 selected genes behaved in the same manner in both *DUSP6* silenced cell lines (Supplementary Figure S2). We conclude the *DUSP6* inhibition affects the expression of proteins involved in substrate anchorage and cell migration through MAPK, TGF-β, and Notch signaling pathway.

### 2.4. DUSP6 Depleted Cells Show Changes in the Organization at Actin Cytoskeleton and Cell-Cell Contact 

**Figure 4:**
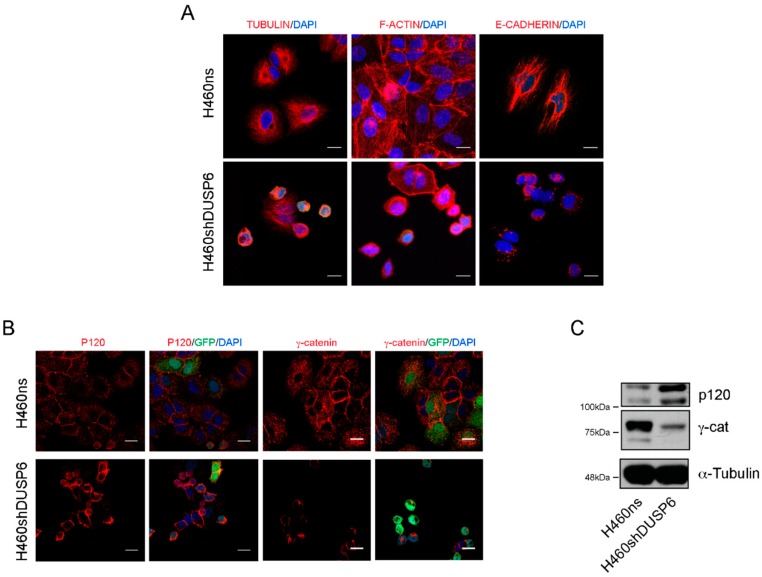
Inhibition of *DUSP6* expression disrupts the interaction cadherin-catenin to the actin filaments and adherent junction. (**A**) Immunofluorescence of tubulin, F-actin and E-cadherin in H460ns and H460shDUSP6 cells. Bar scale corresponds to 100 μm. (**B**) Immunofluorescence of p120 and γ-catenin in H460ns and H460shDUSP6 cells. Bar scale corresponds to 20 μm (**C**) Western blot expression of p120 and γ-catenin in H460ns and H460shDUSP6 cells. Molecular weight is indicated in the left.

The silencing of *DUSP6* gene in NSCLC cells is associated with modification of the expression profile of genes encoding for proteins of the cytoskeleton, as well as proteins involved in cell-substrate adhesion and possibly extracellular matrix remodeling. Microtubules and actin filaments are cytoskeletal protein polymers critical for cell growth and division, motility, signaling and the development and maintenance of cell shape. We have shown that *DUSP6* depleted cells had altered all these parameters (Figure 2). Therefore, we examined actin and tubulin filaments by immunofluorescence in the control and the *DUSP6* silenced cell lines. Firstly, H460shDUSP6 cells revealed a disruption of tubulin network and the actin-stress fibers versus the control cells, as shown by staining with anti-tubulin and F-actin antibodies (Figure 4A). Moreover, immunofluorescence analysis showed decreased E-cadherin expression and abnormal localization out of cell-cell contacts in H460shDUSP6 cells.

Finally, we studied the expression of the cytoplasmic proteins p120 and gamma-catenin (plakoglobin) involved in the attachment of E-cadherin to the actin cytoskeleton. By Western blot analysis in H460shDUSP6 cells showed an increase in p120 protein expression and loss of expression in γ-catenin compared to the control cells (Figure 4C). The distribution of p120 and γ-catenin by immunofluorescence were localized in cell-cell contact in a contiguous manner in the cytoplasm in control cells, while the distribution of p120 and γ-catenin in DUSP6 depleted cells were only around the nucleus (Figure 4B). GFP staining localized in the nucleus in some cells indicated the efficiency of the lentiviral transduction in these cells.

### 2.5. Focal Adhesion Pathway Is Deregulated in Non-Small Cell Lung Cancer Cells Depleted of DUSP6

**Figure 5:**
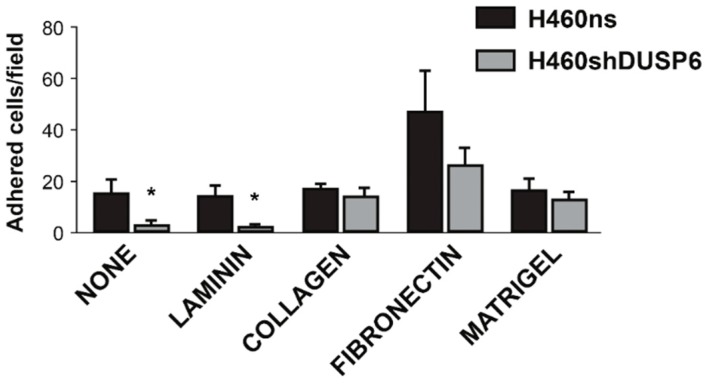
*DUSP6* depletion in H460 cells impaired focal adhesion to the matrix. Wells were coated with laminin, collagen, fibronectin and matrigel or none. H460ns and H460shDUSP6 cells were plated onto matrices and allowed to adhere for 3 h. Non-adherent cells were removed and adhered cells counted. Graph shows the numbers of H460ns and H460shDUSP6 cells adhered onto different matrices per field of view. Statistical significance is indicated by asterisks considering at * *p* < 0.05.

**Figure 6:**
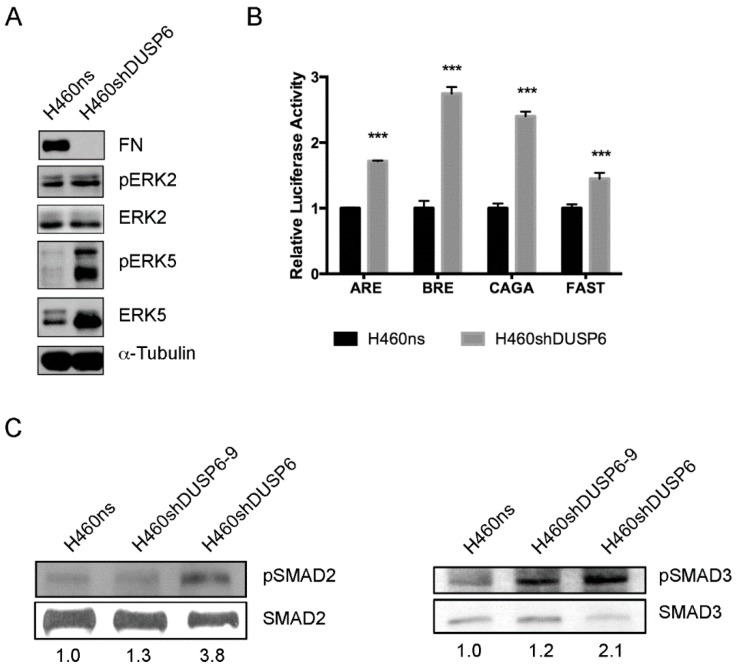
MAPK expression and activation of TGF-β and BMP promoters in H460 cells depleted from *DUSP6*. (**A**) Protein extracts were obtained from H460ns and H460shDUSP6 cells and subjected to Western blot to determine expression levels of fibronectin (FN), ERK5, pERK5, pERK2 and α-tubulin as loading control. (**B**) H460ns and H460shDUSP6 cells were transfected with the corresponding reporter vectors pARE-luc and pFAST-luc as reporters for SMAD2, pCAGA-luc for SMAD3 and pBRE-luc for SMAD1/5. Protein extracts were obtained and luciferase activity was determined for each reporter. Each experiment was performed in triplicate and three different experiments were performed. Statistical significance is indicated by asterisks considering at *** for *p* < 0.001. (**C**) Western blots of phosphorylated SMAD2 and SMAD3 proteins were performed in H460ns, H460shDUSP6 and H460shDUSP6-9 cells. Numbers indicate fold activation estimated by the ratio between phosphorylated and unphosphorylated bands for each SMAD protein.

Based on the previous RNA-seq results, we have found that several genes belonging to the focal adhesion and integrin pathways changed their expression in *DUSP6* depleted cells. Moreover, we observed that H460shDUSP6 cells did not display a good anchorage to the substratum. We set up experiments aimed to define the extracellular matrix requirements for H460shDUSP6 cells. We prepared plates by growing different cell lines and then trypsinizing them in conditions that leave their own extracellular matrix on the plate. Then, after serum depletion of H460shDUSP6 we seeded them in the plates that contained an extracellular matrix layer from H460, H460ns and H460shDUSP6 cells. We observed that H460shDUSP6 cells seeded in plates with extracellular matrix obtained from control (H460 and H460ns) cells, recovered the attachment to the dish with an elongated phenotype (data not shown), but not when the plate contained the matrix from H460shDUSP6 cells. Therefore, it seems that the lack of expression of *DUSP6* in the cells may affect the expression/secretion of ECM proteins. To assess the adhesion capability in H460ns (control) and H460shDUSP6 cells, tissue-culture-treated plates coated with different matrix components (laminin, collagen I, fibronectin and matrigel) were used. The number of cells attached on the plate in the different matrix components were quantified after three hours of seeding. We observed that H460shDUSP6 cells were unable to attach in three hours to laminin matrix and tissue culture treated plates. On the other hand, H460shDUSP6 cells attached as similar efficiency as control cells to matrigel and collagen and with lower efficiency to fibronectin coated plates (Figure 5). When we studied the expression of fibronectin in basal conditions in H460shDUSP6 or in control cells by Western blot, we found that *DUSP6* depleted cells expressed low or undetectable levels of this protein compared to H460ns cells (Figure 6A) but both cell lines expressed the fibronectin gene as detected by qPCR (Figure 2C). This may explain why H460shDUSP6 cells are able to attach to the plates and change their phenotype when fibronectin is supplied externally.

### 2.6. DUSP6 Depletion Activates MAPK and TGF-β Signaling Pathway in Non-Small Cell Lung Cancer Cells

Activation of EGFR is statistically significant and relevant after silencing of *DUSP6* gene expression in NSCLC cells. DUSP6 belongs to a family of MAP kinase phosphatases which inactivate ERK in preference to other activated MAPKs. It is known that the activation of both ERK1/2 and ERK5 pathways can lead to disruption of the actin cytoskeleton [7]. In order to assess which MAPK is activated when *DUSP6* gene expression is inhibited in NSCLC cells, we investigated the phosphorylation of both ERK1/2 and ERK5 proteins under basal growth conditions in both cell lines by Western blotting. *DUSP6* depleted cells showed increased expression and phosphorylation of ERK5, but we could not detect changes in ERK1/2 activation (Figure 6A).

We next investigated the role of DUSP6 in the activation of TGF-β signaling pathway in H460 cells. We used four different reporter constructs for TGF-β and BMP signaling pathways. We used pARE-luc and pFAST-luc as reporters for SMAD2, pCAGA-luc for SMAD3 and pBRE-luc for SMAD1/5. H460ns and H460shDUSP6 cells were transfected with these reporter constructs and we inferred the activation of the *DUSP6* depleted cells compared to control (Figure 6B). The results clearly indicated that the downregulation of *DUSP6* expression activated both reporters for SMAD2, SMAD3 and also for SMAD1/5. We performed a Western blot to confirm the correlation between the activation of the above used promoters and the presence of SMADS phosphorylated forms. Our results showed that two different clones of *DUSP6* depleted cells, H460shDUSP6 and H460shDUSP6-9, showed higher expression levels of pSMAD2 and pSMAD3 (Figure 6C) but not of pSMAD5 (not shown). Those results validated the ones obtained with the pARE-luc, pFAST-luc and pCAGA-luc reporters for SMAD2 and 3 suggesting activation of the TGFβ but not the BMP signaling pathway in *DUSP6*-depleted cells. 

## 3. Discussion

DUSP6 is a dual specific phosphatase localized at the cytoplasm that is usually involved in the inactivation of ERK kinase, and with lower specificity, JNK and p38 kinases. The role of DUSP6 in cancer is mostly related with the type of tumor, being pro-oncogenic or tumor suppressive. Previous work in the literature described it as an ERK-mediated inhibitor in lung cancer cells [4], or to be required for tumor associated angiogenesis. However, the lack of studies in large databases comparing the expression of *DUSP6* with survival in lung cancer has not allowed a clear picture on the role of DUSP6 in this type of tumor. Here, we present results obtained from two different databases GSE4537 [11] and LUAD TCGA indicating a good correlation between low *DUSP6* expression and poor outcome of lung adenocarcinoma patients. Interestingly, no correlation was obtained in LUSC in TCGA. It has been published, that in LUAD, 15–20% mutations belonged to the EGFR pathway where DUSP6 plays an active role. On the contrary in the carcinogenesis pathways implicated in LUSC, mutations occur in the cell cycle, p53 and the PI3K-Akt signaling pathway [12]. These data suggest that *DUSP6* could be a tumor suppressor in lung adenocarcinoma. 

We have also studied which parameters related with tumorigenicity and tumor progression would be affected by low expression levels of DUSP6. Firstly, *DUSP6* depleted cells showed important changes in morphology, growing as rounded cells and detached very easily from the plate. We observed that *DUSP6* depleted cells presented a higher motility which would agree with the acquisition of metastatic capacity. Expression of several genes has been related with the increase in metastatic properties including those of *SNAIL2, ZEB1 SNAIL1* [13,14]. H460shDUSP6 cells expressed higher levels of these three genes and may justify changes in motility and cell shape found in *DUSP6* depleted cells. We also found an increase in the tumorigenic capacity in H460shDUSP6 cells. The total number of tumors was higher in mice injected with H460shDUSP6 cells than in mice injected with H640ns cells (100% vs. 71% respectively) and the number of tumors induced was bigger in H460shDUSP6 cells (50% vs. 20%) which is in accordance with poor prognosis in NSCLC patients with low levels of *DUSP6* expression.

The analysis of differentially expressed genes performed in H460shDUSP6 vs. H460ns cells showed upregulation of genes involved in cellular functions compatible with higher aggressiveness of *DUSP6* depleted cells. Cellular functions such as focal and integrin-mediated adhesion or actin cytoskeleton regulation and pathways regulated by EGF/EGFR, TGFß, WNT, interleukin signaling, Notch and growth factors are represented by many genes whose expression levels changed among both cell lines. Several genes belonging to those pathways were overexpressed in *DUSP6* depleted cells. One of them, Tenascin-C expression is upregulated and has been involved in gastric [15] and gastric GIST cancer progression [16], increased proliferation in pancreatic cancer [17] and is a prognostic determinant of colorectal cancer through induction of epithelial to mesenchymal transition and proliferation [18]. *CCND2* gene has been shown to be targeted by expression of mir-4317 and miR-671-3p and related to inhibition of cancer progression in NSCLC [19]. *VAV3* upregulation (Rho exchange factor) has been described in lung cancer metastasis and breast cancer. miR-499-5p inhibits NSCLC proliferation and metastasis by targeting *VAV3* [20]. *RUNX2* and *LEF1* are also targeted by miR-196b [21] and miR-557 [22] inhibiting lung cancer metastasis. *FGFR2* is described as a prognostic marker of recurrence in lung adenocarcinoma [23]. BMP4 depletion by miR-200 inhibits tumorigenesis and metastasis in lung adenocarcinoma cells [24] and finally *FYN* promotes cancer progression through epithelial-to-mesenchymal transition [25]. All of these genes were upregulated in our H460shDUSP6 depleted cells suggesting DUSP6 is tackling different pathways involved in tumor progression such as EMT, ERK, TGFβ and WNT.

Some selected genes from RNA-seq data were validated by qPCR in both H460shDUSP6 and H460shDUSP6-9 clones. Furthermore, we observed similar changes in expression of *CAPN6, CCND2* and *LEF1* genes in *DUSP6* depleted A549 cells, indicating that the inhibition of *DUSP6* in other NSCLC cell lines affects the expression of the same genes. In agreement with the RNA-seq analysis, DUSP6 depleted cells had a disruption of tubulin network and actin-stress fibers. Moreover, the lack of expression of E-cadherin in H460-DUSP6 depleted cells should be responsible of the lack of attachment to the actin cytoskeleton. We observed that the expression of the cytoplasmic proteins p120 and γ-catenin distributed only around the nucleus and not cell-cell contact sites in a contiguous manner as it should be. All together the results indicated that the inhibition of *DUSP6* protein expression in NSCLC affects the interaction cadherin–catenin complexes at the adherent junctions. *DUSP6* depleted cells did not express fibronectin when assessed by Western blot. As a consequence, *DUSP6* depleted cells attached very loosely to the plate. Studies with different substrates showed that H460shDUSP6 cells recover the morphological appearance of control cells when fibronectin and laminin were coated in the plate. Accordingly, qPCR revealed that *SNAI1, SNAI2* and *ZEB1* are overexpressed in *DUSP6* depleted cells and these transcription factors can repress E-cadherin expression. We also found that H460shDUSP6 showed increased levels of phosphorylated ERK5, but surprisingly not increased levels of phosphorylated ERK1/2. Therefore, the morphological changes observed in *DUSP6* depleted cells may be related to the activation of ERK5 [26].

In contrast by the role of DUSP6 as a tumor suppressor that we describe here pharmacological inhibition of DUSP6, suppresses gastric cancer growth and metastasis and overcomes cisplatin resistance [27]. This dual role of tumor suppressor proteins has been described for other classical tumor suppressors such as p21 (*CDKN1A*) and p14ARF. Both proteins can also act as prooncogenic. P21 chronic expression in response to p53 activation causes genome instability [28] and p14ARF has been described as mutated in different types of tumors such as colorectal, gastric carcinomas, melanoma and glioblastoma [29]

It has been described that TGF-β acts as a potent inducer of EMT by combining both SMAD and non-SMAD signaling pathways and directly activating the expression of the EMT transcription factors, SNAIL and SLUG, ZEB1 and ZEB2, and TWIST [27,30,31]. Interestingly and in agreement with the changes in motility and tumorigenicity, our results revealed upregulation in TGF-β signaling pathway in our RNA-seq, probably mediated by SMAD2 and 3 activation in *DUSP6* depleted cells. 

## 4. Materials and Methods

### 4.1. Cell Culture, Compounds and Luciferase Assay

The human non-small cell lung cancer cell line H460 and A549 were purchased from the American Type Culture Collection (ATCC) and maintained in RPMI medium supplemented with 10% FBS. Puromycin was purchased from Sigma-Aldrich (St. Louis, MO, USA). 

For luciferase assays, pARE-luc and pFAST-luc as reporters for SMAD2, pCAGA-luc for SMAD3 and pBRE-luc for SMAD1/5 (250 ng) and control Renilla reporters (5 ng) were transfected into H460ns and H460shDUSP6 cells using Lipofectamine 2000 transfection reagent (Invitrogen, Carlsbad, CA, USA). The relative luciferase activity was determined after 24 h of transfection using the Dual-Luciferase Reporter Assay System (Promega, Madrid, Spain) and normalized to protein concentration measured using Bradford reagent (Bio-rad laboratories, Hercules, CA, USA). Results are average of three independent experiments (each sample is by triplicate in each experiment) and expressed as mean values ± standard deviation (s.d.).

### 4.2. Lentivirus Production and Cell Infections

GPZ Lentiviral vectors containing shRNAmir for human DUSP6 (RHS4430-99889522 and RHS4430-101132249) were purchased from Open Biosystems. These vectors contain a GFP reporter which allows to mark the cells expressing the shRNA and a puromycin drug resistance for selection of stable cell lines. Lentiviral supernatants were produced in HEK293T cells using a viral packaging system that includes the (VSVg) pMD2.G and pCD-NL-BH plasmids (kindly provided by Juan Bueren) as described in [32]. Two and three days after transfection, viral supernatants were collected and used to infect and re-infect H460 and A549 cells. Transfected cells were then selected by adding 2 µg/mL puromycin for ten days. The empty vector pGIPz-shARN^mir^-NS was used as a control of infection. All lentiviral selected clones were analyzed for expression of *DUSP6* by qPCR and the clones exhibiting minimal expression for *DUSP6* were selected. Two independent pools per gene were generated. 

### 4.3. RNA-seq Studies

RNA was isolated using the TriReagent (Sigma-Aldrich) according to the instructions provided by the manufacturer. Quantity and quality of the RNA was assessed using the Bioanalyzer 2100 (Agilent Technologies, Santa Clara, CA, USA) and Qubit 2.0 (Life Technologies, Carlsbad, CA, USA) system. Poly(A)+ mRNA was isolated from 3 µg of total RNA using Oligo-T-attached magnetic beads (Illumina, San Diego, CA, USA), chemically fragmented and converted to cDNA by reverse transcription. cDNA fragments were purified and amplified to create a double-stranded cDNA library following the manufacturer’s instructions (Illumina). Sequencing of the libraries was made by paired-end reactions (100 × 2) using an Illumina HiSeq2000 Sequencer. The sequences generated were mapped to the human genome program http://www.ensembl.org. Transcripts were identified and quantified using the software cufflinks v2.02 [33]. The number of reads per gene was determined with the HTSeq Package (http://www-huber.embl.de/users/anders/HTSeq). Statistical analysis and identification of genes differentially expressed were made using the softwares EDASeq (http://www.bioconductor.org/packages/2.11/bioc/html/EDASeq.html) and DESeq [34]. A minimal difference of two times and an FDR [35] of 0.05 were considered. Functional enrichment analysis was based on Pathways Database (http://www.wikipathways.org/index.php/WikiPathways), considering a minimal FDR of 0.05.

### 4.4. Validation of mRNA Changes by RT-q-PCR

Eight genes were found to be differentially expressed in the RNA-seq studies (*CCND2, COL3A1, FYN, RUNX2, TNC, BMP4, CAPN6* and *VAV3*). These genes were selected and their expression validated by real-time RT-PCR. 1 µg of total RNA extracted from H460 and A549 cells was retrotranscribed using a High-Capacity cDNA Archive Kit (Applied Biosystems, Foster City, CA, USA) to produce cDNA. Reverse transcription was performed at 25 ºC for 10 min and 37 °C for 2 h. Then, each cDNA sample was analyzed in triplicate using the ABI PRISM 7700 Sequence Detector (PE Applied Biosystems). Real-time PCR was carried out using Taqman Universal PCR Master Mix (Applied Biosystems), containing ROX to normalize emissions. 

Primers used for amplification of the eight selected genes were purchased from Applied Biosystems as Taqman Gene Expression Assays as follows, Assay ID: *DUSP6* Hs00737962m1, *CCND2* Hs00153380_m1, *COL3A1* Hs00943809_m1, *FYN* Hs00176628_m1, *RUNX2* Hs01047973_m1, *TNC* Hs01115665_m1, *BMP4* Hs03676628_s1, *CAPN6* Hs00560073_m1, *VAV3* Hs00916818_m1 and *β-ACTIN* Hs01060665_g1. The following thermal cycling conditions were applied: 10 min at 95 °C (1 cycle), 15 s at 95 °C and 1 min at 60 °C (40 cycles).

Relative gene expression quantification was calculated according to the comparative threshold cycle method (2^−ΔΔ*C*t^) [36] using *β-ACTIN* as endogenous control. Normalized expression values were determined as follows: 2^–(Δ*C*t sample − Δ*C*t calibrator)^, where Δ*C*t values were calculated by subtracting the *C*t value of the target gene from the value of the endogenous control gene RQ (Relative quantification, [36]).

### 4.5. Evaluation of Gene Expression of Epithelial to Mesenchymal Transition Associated Genes by RT-q-PCR

Total RNA extracted from H460 and A549 cells was retrotranscribed as indicated in previous paragraph. Real-time PCR was carried out using qPCR^TM^ core kit for SYBR^TM^ Green I (Applied Biosystems) according to the manufacturer’s instructions. *GAPDH* was used as an endogenous control for the relative quantification. The specific primers were as Table 1.

### 4.6. Western Blots

Whole-cell extracts from H460 cells were prepared using cell lysis buffer containing 25 mM HEPES pH 7.5, 0.3 mM NaCl, 1.5 mM MgCl_2_, 0.2 mM EDTA, 0.5 mM DTT, 20 mM β-glycerophosphate, 0.1 mM Na_3_VO_4_, 0.1% Triton X-100 and complete protease inhibitor cocktail (P8340 Sigma, Madrid, Spain). Lysates were centrifuged and the supernatants transferred to a new tube. After adding SDS loading buffer the lysate was boiled for 5 min. 20 µg of protein extract was separated on SDS-polyacrylamide gel and electroblotted to Immobilon P membranes (Millipore, Burlington, VT, USA). Antibodies used were anti-p-ERK5 (3371, Cell Signaling, Danvers, MA, USA), anti-ERK5 (3372, Cell Signaling), anti-p-ERK1/2 (9106, Cell Signaling), anti-fibronectin (sc-71113, Santa Cruz Biotechnology, Dallas, TX, USA), anti-p120 (P17920, BD Biosciences, Franklin Lakes, NJ, USA), γ-catenin (610254, BD Transduction, Franklin Lakes, NJ, USA), anti-β-tubulin (T9026, Sigma, St. Louis, MO, USA), phospho-Smad2/3 (Ser 423/425) and Smad2/3 (FL-425) were from Santa Cruz Biotechnology (Dallas, TX, USA). The signal was detected using enhanced chemiluminiscent (ECL) method (Santa Cruz Biotechnology). After washes the blots were re-incubated with the α-tubulin antibody to normalize for protein load. 

### 4.7. Cell Motility Assay 

H460ns and H460shDUSP6 cells were plated at 3 × 10^5^ density on six multiwell dishes. The next day the culture medium was changed to eliminate debris, and live-cell imaging was carried out using time-lapse microscopy with a Cell Observer Z1 (Zeiss, Oberkochen, Germany) (at 37 °C and 5% CO_2_/95% air) coupled to a Cascade 1 k camera. Cells were imaged for 24 hours at 30 minutes intervals. Images were processed using AxioVision 4.8 imaging software and individual cell movement was analyzed with Image J software. Motility assays were performed a minimum of three times, analyzing at least 50 cells observed per experiment.

### 4.8. Immunofluorescence 

Cells were grown onto 8 mm diameter coverslips, transfected and fixed in 3.7% formaldehyde solution (47608, Fluka, Loughborough, UK) at room temperature for 15 min. After washing with PBS, cells were permeabilized with 0.2% Triton X-100 and blocked with 10% horse serum to prevent non-specific staining. The primary antibody was then added to the blocking buffer and the cells incubated overnight at 4 °C. Primary antibodies: Mouse anti-γ-catenin (610254, BD Transduction), mouse anti–p120 (P17920, BD Biosciences), mouse anti-α-tubulin (T9026, Sigma-Aldrich), and E-cadherin (ECCD2, Zymed laboratories). The day after, the cells were washed in PBS with 0.1% Triton X-100 for three times and incubated with Alexa 546 goat anti-mouse, Alexa 647 goat anti-mouse, (Molecular Probes, Eugene, OR, USA) fluorophore conjugated secondary antibodies for signal detection. The actin filaments were stained using Alexa 546-conjugated phalloidin (Molecular Probes) added after the blocking step. After washes in PBS with 0.1% triton, coverslips were counterstained with 4′,6-diamidino-2-phenylindole (DAPI) and mounted in Prolong Gold Antifade Reagent (Molecular Probes). The micrographs were obtained at Confocal Spectral Leica TCS SP5 and LAS-AF 1.8.1 Leica software.

### 4.9. Adhesion Assays

Six well tissue culture plates containing coverslips were coated with one of the following adhesion matrices: Human fibronectin (ThermoFisher, Waltham, MA, USA), mouse laminin (Sigma-Aldrich), rat tail collagen I (ThermoFisher), or matrigel, (BD Biosciences) overnight, according to the manufacturer’s recommendations. One well was left uncoated as a control. After a wash in PBS cells were seeded onto the plates at 3 × 10^5^ density in serum free culture medium (RPMI) and incubated for 3 h at 37 °C. The cells were finally washed in PBS to remove any unattached cells, fixed and analyzed by immunofluorescence as described in the previous section. The number of adhered cells was determined by counting ten random fields per sample. The experiments were run in triplicate.

### 4.10. Tumorogenicity in Xenograft Mouse Model

*nu/nu* mice were obtained from Charles River Laboratories (Wilmington, MA, USA). In order to satisfy statistical constraints, twenty-eight 6-week old mice were injected subcutaneously with one million H460ns or H460shDUSP6 cells embedded in matrigel in each flank. Tumor measurements were recorded since visible tumor appeared. The tumor volume was calculated by measuring the two longest axes in the x/y plane with a vernier caliper and using the following formula: Tumor volume = (length × width^2^) × 0.52. 19 days after the cells were injected; mice were sacrificed. 

### 4.11. Statistical Analysis

For the statistical analysis of the results, the mean was taken as the measurement of the main tendency, while standard deviation was taken as the dispersion measurement. *t*-Student was performed. The significance has been considered at * *p* < 0.05. ** for *p* < 0.01 and *** for *p* < 0.001. GraphPad Software v5.0 was used for statistical analysis and graphic representations.

### 4.12. “In Silico” Data

To validate the role of DUSP6 as tumor suppressor, some independent lung microarray and related clinical data sets of NSCLC series were obtained from Gene Expression Omnibus (GEO) database [11] and The Cancer Genome Atlas (TCGA) lung adenocarcinoma raw data available at https://tcga-data.nci.nih.gov/tcga/dataAccessMatrix.htm?mode=ApplyFilter&showMatrix=true&diseaseType=LUAD. Then statistical significance in survival time from the data “in silico” were estimated according to the method of Kaplan–Meier. Survival distributions considering tertiles (33%) of data were determined using the log-rank test. *p* < 0.05 was considered to have statistical significance.

## 5. Conclusions

**Figure 7:**
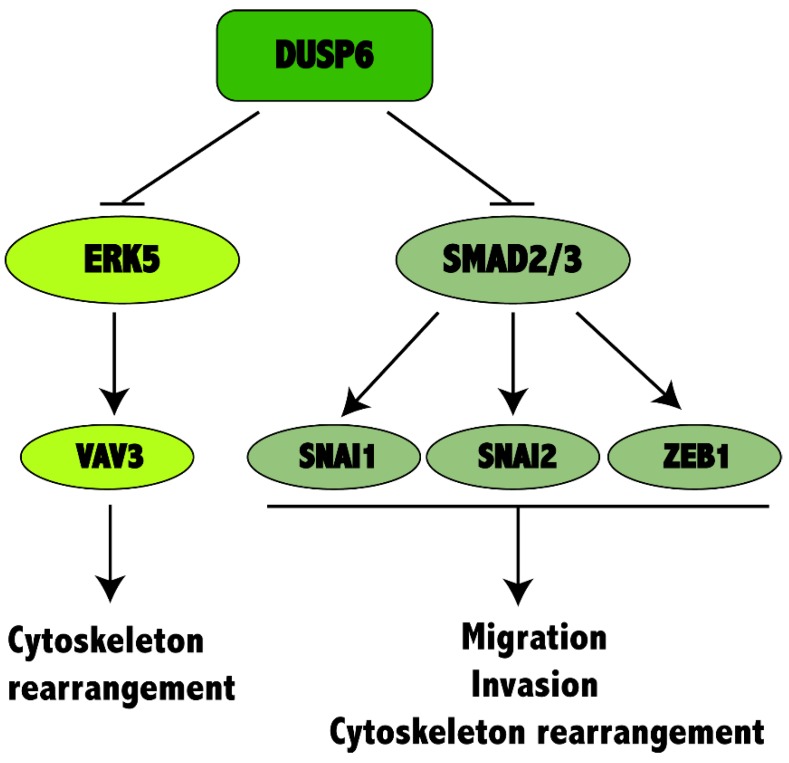
DUSP6 activity on NSCLC cancer progression. If *DUSP6* is expressed, it inhibits the activity of ERK5 and the expression of SMAD2/3 is repressed, therefore inhibiting changes in cystoskeleton organization, migration and invasion. When *DUSP6* expression is inhibited in NSCLC, ERK5 phosphorylation increases and also SMAD2/3 expression, thus activating the expression of *SNAI1, SNAI2, ZEB1*, among others involved in migration, invasion and inducing cytoskeleton rearrangement.

Altogether our results suggest that DUSP6 maintains a transcriptional program that represses EMT and tumorigenicity by downregulating ERK5 phosphorylation, inhibiting of EGF/EGFR, TGF β, WNT and interleukin signaling pathways involving *VAV3, SNAI1, SNAI2 and ZEB1*, among others, hence acting as a tumor suppressor. Low expression levels of *DUSP6* in NSCLC would affect these pathways by increasing tumorigenicity, cell motility, EMT transition and consequently tumor progression associated with poor prognosis in NSCLC patients. Our manuscript sheds light on the unclear mechanism of downregulation of *DUSP6* expression in NSCLC and other types of tumors and strongly indicates that re-establishment of *DUSP6* expression seems to be a good strategy to revert the malignant phenotype (Figure 7).

## Figures and Tables

**Table 1:** Primers used for real-time PCR.

Gene	Forward Primer	Reverse Primer
*SNAI1*	5′ ACCCACACTGGCGAGAAG 3′	5′ GAGAAGGATGTGGGGTCCTT 3′
*SNAI2*	5′ TGGTTGCTTCAA GGACACAT 3′	5′ GTTGCAGTGAGGGCAAAGAA 3′
*FN1*	5′ CCCTTACAGTTCAGGGTTCC 3′	5′ TTCAAGCCTTCGTTGACAGA 3′
*VIM*	5′ GACAATGCGTCTCTGGCACGT 3′	5′ TCTTCTGCCTCCTGCAGGTTCTT 3′
*ZEB1*	5′ GCTGACCAGAACAGTGTT 3′	5′ CAGAGTCATTCTGATCCTC 3′
*GAPDH*	5′ GAGAGACCCTCACTGCTG 3′	5′ GATGGTACATGACAAGGTGC 3′

## Supplementary Materials

Supplementary materials can be found at https://www.mdpi.com/1422-0067/20/8/2036/s1.

## Abbreviations

ATCC	American Type Culture Collection
ECACC	European Collection of Cell Cultures
NSCLC	Non-Small Cell Lung Cancer
MAPKs	Mitogen-activated Protein Kinases
ESCC	Esophageal squamous cell carcinoma
NPC	Nasopharyngeal carcinoma
ECM	Extracellular matrix
GIST	Gastrointestinal stromal tumors
FDR	False Discovery rate
ROX	(6-Carboxyl-X-Rho-damine
