# Supplementary material for: Role of Dusp6 Phosphatase as a Tumor Suppressor in Non-Small Cell Lung Cancer
Source: Int J Mol Sci. 2019 Apr 25;20(8):2036. doi: 10.3390/ijms20082036 (PMC6514584; doi:10.3390/ijms20082036)
Supplement: Supplementary file 1 [file ijms-20-02036-s001.zip › ijms-472059-supplementary.pdf]

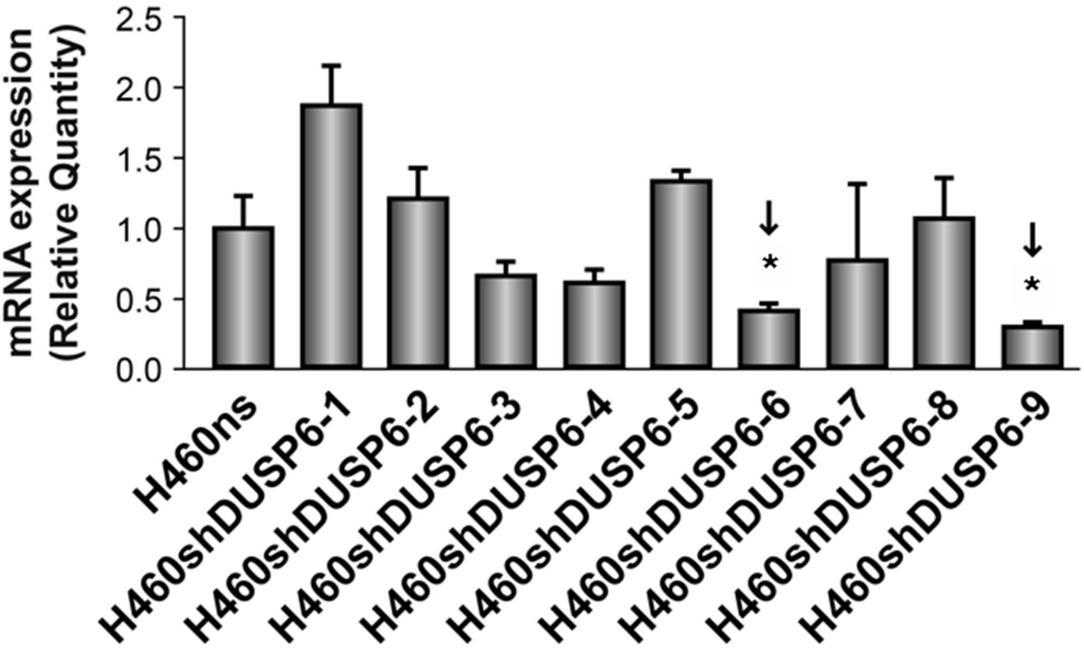

Supplementary Figure S1: Expression of DUSP6 in H460 cells transduced with 9 different shRNAmir for human DUSP6. H460 cells were transduced with nine lentiviral vectors described in Materials and Methods and 9 different clones were obtained and named H460shDUSP6-1 to H460shDUSP6-9. Expression levels of DUSP6 were determined by RT-QPCR using H460ns as a reference and GAPDH as control for expression. Statistical significance indicated by asterisks

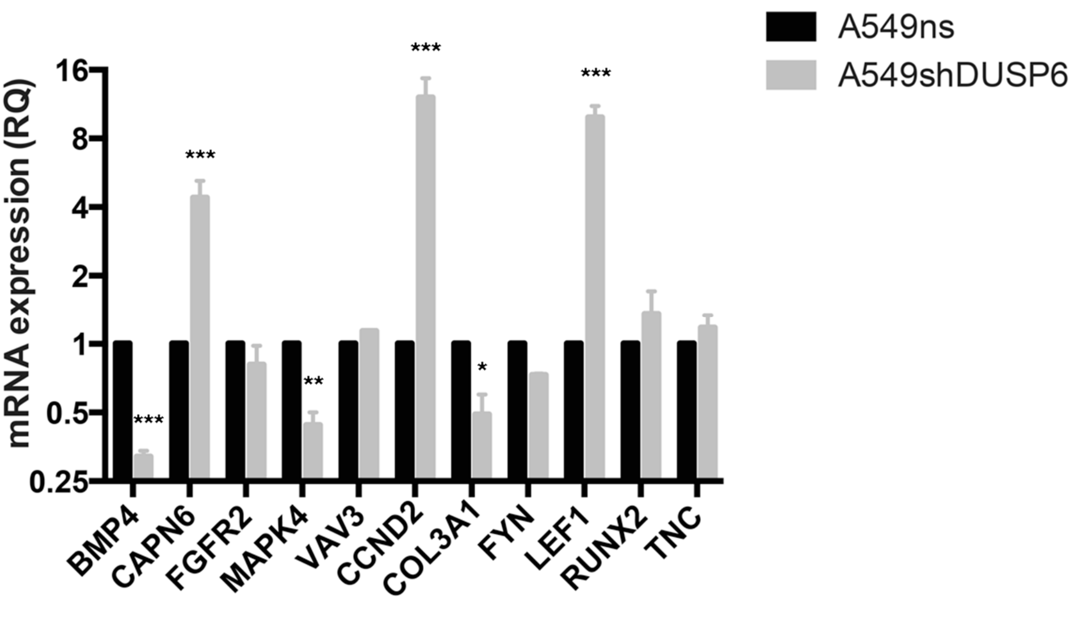

Supplementary Figure S2. RNA-seq validation by qRT-PCR in DUSP6 depleted A549 cells, of selected genes (in bold genes Table B). Data were normalized to *β-actin* levels and are shown as mean± s.d. of three independent experiments. Statistical significance indicated by asterisks
